# Supplementary material for: Characterization and Pathogenicity of Two Novel PRRSVs Recombined by NADC30-like and NADC34-like Strains in China
Source: Viruses. 2022 Sep 30;14(10):2174. doi: 10.3390/v14102174 (PMC9607012; doi:10.3390/v14102174)
Supplement: Supplementary file 1 [file viruses-14-02174-s001.zip › viruses-1935189-supplementary.pdf]

Supplementary Figure

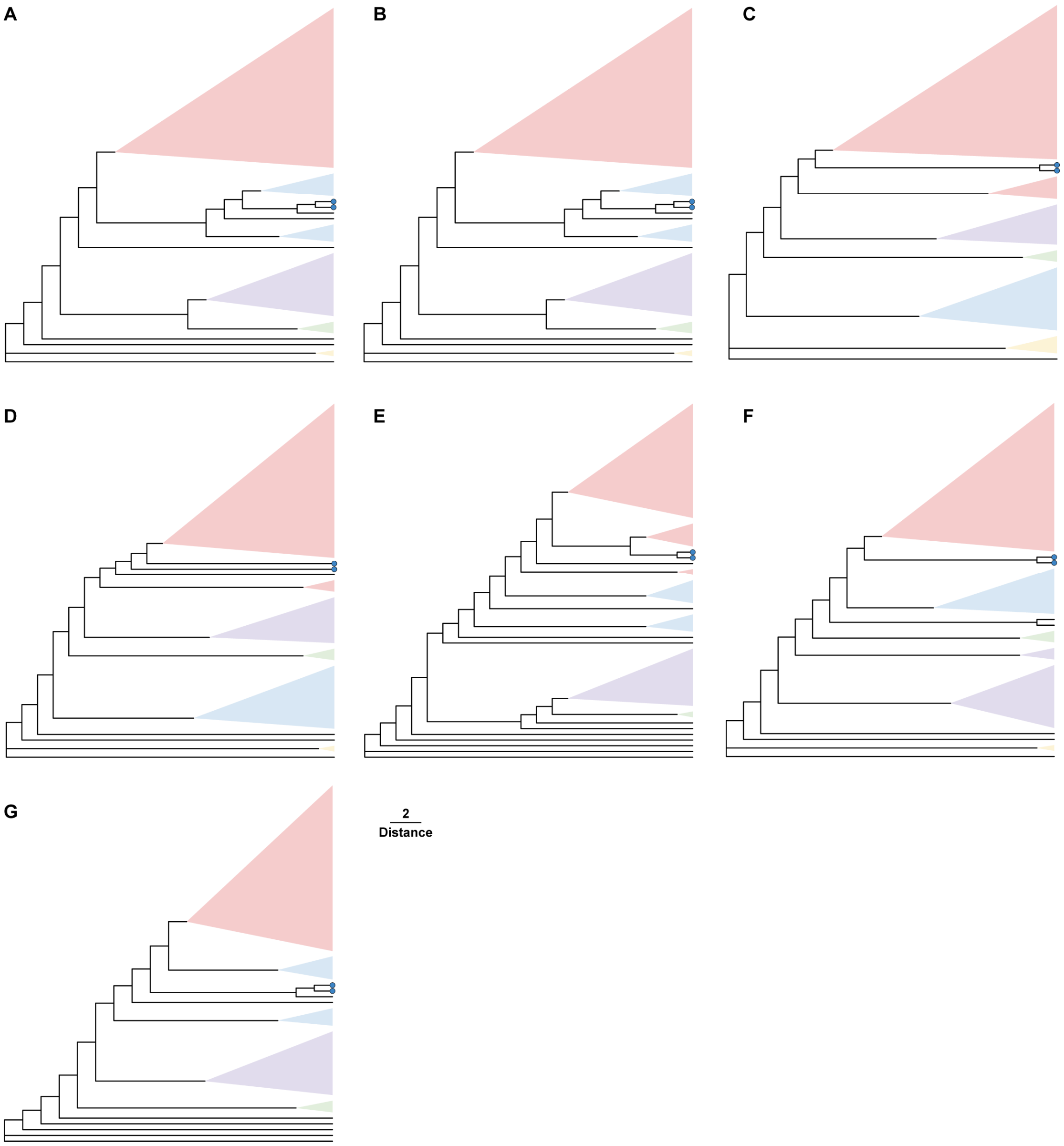

Supplementary Figure S1. (A-G): Phylogenetic trees constructed based on the ORF1a, 1b, 2, 3, 4, 6 and 7 gene of GD-H1 and GD-F1 strains with 61 reference PRRSV strains.
